# Immune dysfunction in COVID-19 and judicious use of antirheumatic drugs for the treatment of hyperinflammation

**DOI:** 10.3906/sag-2110-179

**Published:** 2021-12-17

**Authors:** Abdurrahman TUFAN, Marco MATUCCI CERINIC

**Affiliations:** 1 Division of Rheumatology, Department of Internal Medicine, Gazi University, Ankara Turkey; 2 Division of Rheumatology AOUC, Department of Experimental and Clinical Medicine, University of Florence , Florence Italy; 3 Unit of Immunology, Rheumatology, Allergy and Rare Diseases (UnIRAR), IRCCS San Raffaele Hospital, Milan Italy

**Keywords:** SARS-CoV-2, COVID-19, inflammation, cytokine storm, treatment, rheumatology

## Abstract

In the Wuhan province of China, almost two years ago, in December 2019, the novel Coronavirus 2019 has caused a severe involvement of the lower respiratory tract leading to an acute life-threatening respiratory syndrome, coronavirus disease-19 (COVID-19). Subsequently, coronavirus 2 (SARS-CoV-2) rapidly spread to the entire world causing a pandemic and affected every single person on earth either directly or indirectly with destroying all facets of social life and economy. Since the announcement of COVID-19 as a global pandemic, we have witnessed tremendous scientific work on all aspects of COVID-19 across the globe, which has never been witnessed before. The most remarkable achievement would be the introduction of vaccines, which provide protection from the severe infection and is the only premise for the control of disease. However, despite the tremendous work, the number of treatments either antiviral or immunomodulatory for infected patients are considerably limited, yet disease is causing substantial morbidity and mortality. COVID-19 follows heterogenous disease course among infected individuals, and dysregulated immune system is primarily responsible for the worse outcomes. Immune deficiency, being on corticosteroids for inflammatory diseases, delayed interferon response and advanced age adversely influence prognosis with impairing viral clearance. On the other hand, exuberant immune response with features of cytokine storm is the leading cause of death, which can be alleviated by use of either general immunosuppression with corticosteroids or selective neutralization of potent pro-inflammatory cytokines such as interleukin (IL)-1 and IL-6. Herein, we summarized the potential effective immunomodulatory treatments emphasizing in which patient population it is the most suitable, which dose should be administered, and which is the most appropriate timepoint to administer the drug during the course of the disease.

## 1. Introduction

In December 2019, almost two years ago, in the Wuhan province of China, the novel Coronavirus 2019 (COVID-19) has caused an outbreak with severe involvement of the lower respiratory tract leading to a new acute life-threatening respiratory syndrome. Subsequently, coronavirus 2 (SARS-CoV-2) rapidly expand to other continents causing a pandemic, which affected every single person on the earth either directly or indirectly with destroying all facets of social life and economy. Since the announcement of COVID-19 as a global pandemic, we have witnessed tremendous scientific work on all aspects of COVID-19 across the globe, which has never been witnessed before. The most remarkable achievement would be the introduction of vaccines, which provide protection from severe infection and is the only premise for the control of the disease. However, despite the tremendous work, the number of treatments either antiviral or immunomodulatory for infected patients is considerably limited, yet the disease is causing substantial morbidity and mortality. 

COVID-19 follows a heterogenous disease course among infected individuals and dysregulated immune system is primarily responsible for the worse outcomes [1]. According to the disease progression, patients may be roughly divided into two groups; asymptomatic or mild cases that usually recover and severe cases (approximately 15%) that develop serious lung inflammation, acute respiratory distress syndrome (ARDS), cardiac and renal injury, multiorgan failure and thromboembolic events, especially in patients with older age, comorbidities, and yet little known genetically susceptible individuals [2–5]. Immune deficiency, being on corticosteroids for inflammatory diseases, delayed interferon response, and advanced age are adverse prognostic factors that impair viral clearance [6]. On the other hand, exuberant immune response with features of Cytokine Storm (CS) or Multisystem Inflammatory Syndrome (MIS-) is the leading cause of death which can be alleviated by use of either general immunosuppression with corticosteroids or selective neutralization of potent pro-inflammatory cytokines such as interleukin (IL)-1 and IL-6. However, neither approach has achieved high clinical success rates in patients with severe COVID-19 necessitating urgent development of more effective agents. 

An efficient immune response against SARS-CoV-2 may be considered fundamental for the resolution of COVID-19. However, studies have shown a significant relationship between the disease severity and the dysregulated immune system, including exhausted T cell response, lymphopenia, neutrophilia, dysregulation (overactivation) of macrophages, impaired type I interferon (IFN-I) response, antibody-dependent enhancement, and especially, cytokine storm (CS) [3, 7]. It has been suggested that, during the response to SARS-CoV-2, the immune dysregulation and the high level of pro-inflammatory cytokines are the main cause of tissue injury. Eventually, the exact immunopathogenesis of COVID-19 remains to be elucidated, but, in brief, overactivated innate and impaired adaptive immune responses characterize severe COVID-19.

The main challenge at current is the identification of patients who would progress into critical disease and whether a specific subset of patients might benefit most from the immunomodulatory treatments. Several well-designed clinical trials demonstrated the efficacy of certain drugs in COVID-19 that are used in daily rheumatology practice, including corticosteroids, tocilizumab, anakinra, Janus Kinase (JAK) inhibitors, colchicine, and intravenous immunoglobulin. Herein, we discussed altered immune responses in COVID-19 patients and current treatment options for severe COVID-19, particularly emphasizing the rational use of drugs, on the basis of either the timing of the therapy, relevant inflammatory characteristics of the patient, or both. 

## 2.The origin and structural features of SARS-CoV2

SARS-CoV-2 has nonsegmented, single-stranded positive-sense RNA (+ssRNA) with 5’-cap structure and 3’-poly-A tail, which is the typical genomic structure of the betacoronaviruses like SARS-CoV and MERS-CoV [8]. The genome of CoV contains six major open reading frames (ORFs) and numerous accessory genes. First ORFs (ORF1a/b), which encompasses the two-third of viral RNA, encode two large proteins of CoVs, polyprotein 1a (pp1a) and pp1ab. These polyproteins are divided into 16 nonstructural proteins (nsps), responsible for viral RNA replication and transcription, by virally encoded chymotrypsin-like protease (3CLpro) or main protease (Mpro) and papain-like protease (PLpro) [9, 10]. The remaining OFRs on the one-third of the genome encode major structural proteins, including spike (S), envelope (E), membrane (M), and nucleocapsid (N) proteins, all of which are crucial for the viral infectivity. CoVs possess a lipid bilayer envelope with S, M, and E proteins. 

Viral N protein acts as an antagonist to the interferon pathway by regulating the signaling and synthesis of type I interferon (IFN), which is one of the most critical responses in the innate immunity to viral infection [11]. The M protein is the most abundant component of the viral envelope that gives the shape of the virus and promotes the membrane curvature and the virus assembly by interacting with the S protein and the ribonucleoprotein [12–14]. The E protein is a small integral membrane protein that facilitates the assembly, the budding, and the envelope formation as well as the M protein [15]. Moreover, the E protein has an ion-channel activity and activates inflammasomes [16].

The S glycoproteins on the surface of CoVs are the receptor binding proteins, which are responsible for the attachment to host cells, viral-host cell membrane fusion, and the internalization of the virus. The S glycoprotein consists of two domains: S1 domain, which includes receptor-binding domain (RBD), interacting with angiotensin converting enzyme 2 (ACE2) on the human host cells as SARS-CoV, and S2 domain, which mediates virus-cell membrane fusion and viral entry [17, 18]. After attachment of SARS-CoV-2 with S protein to ACE2 on the host cells, S protein is cleaved by host cell proteases to reveal the S2 domain for viral-host membrane fusion and viral entry, which is coupled with TNF-α production [8, 19, 20]. 

## 3. The immune response and cytokine storm in COVID-19

The effective antiviral responses of the host innate and adaptive immunity, including the production of various proinflammatory cytokines, the activation of T cells, CD4 and CD8+ T cells, are essential for inhibition of viral replication, resolution of virus-induced inflammation, and clearance of infected cells [21, 22]. Nevertheless, the tissue injury caused by the virus could stimulate abnormal production of proinflammatory cytokines, the recruitment of proinflammatory macrophages and granulocytes. This results in the CS with shared features with macrophage activation syndrome (MAS) or secondary hemophagocytic lymphohistiocytosis (sHLH), thus leading to extensive tissue damage [23–25]. Data obtained from SARS-CoV-2 infected patients have shown that severe cases may be characterized by a cytokine storm inexorably progressing to ARDS [26–29]. Several features of COVID-19, such as the cytokine profile, serological markers, and clinical symptoms, resemble viral infection triggered sHLH [3, 24]. Furthermore, severity of COVID-19 is closely associated with the level of the proinflammatory cytokines and subsets of immune cells [3, 30].

COVID-19 possesses different levels of various cytokines and chemokines through the mild to severe stage of the disease. The retrospective analysis has demonstrated that initial plasma levels of IL-1β, IL-1RA, IL-7, IL-8, IL-10, IFN-ɣ, monocyte chemoattractant peptide (MCP)-1, macrophage inflammatory protein (MIP)-1A, MIP-1B, granulocyte-colony stimulating factor (G-CSF), and tumor necrosis factor-alpha (TNF-α) are increased in patients with COVID-19. The further analysis has shown that the plasma concentrations of IL-2, IL-7, IL-17, IL-10, MCP-1, MIP-1A, and TNF-α in intensive care unit (ICU) patients are higher than that of non-ICU patients [26]. Moreover, the plasma levels of IL-2, IL-6, IL-8, IL-10, and TNF-α, observed in severe infection, are prominently greater than those with nonsevere infection [27]. Few retrospective studies have revealed that the lung injury is strongly associated with the levels of IL-1α, IL-1ra, IL-2, IL-7, IL-10, IL-17, IFN-ɣ, inducible interferon protein (IP)-10, G-CSF, and MCP-3 and all of these cytokines and chemokines excluding MCP-3 are positively related to SARS-CoV-2 viral load [7, 28]. The plasma level of IL-6, a notorious cytokine contributing to MAS, increases both in mild and severe patient groups of COVID-19 with being remarkably higher among the latter group of patients [3, 27, 29, 31]. Furthermore, based on the assessment of pulmonary infiltration in patients with ARDS, the large area of lung injury (≥ 50%) is closely correlated with the increased level of IL-6 and the subgroup of lymphocytes in the peripheral blood [32]. 

During the infection, both innate and adaptive immune cells synergistically participate in the antiviral response [33]. The important increment in the number of neutrophils, leukocytes, and the neutrophil-lymphocyte-ratio (NLR) has been observed in severe COVID-19 compared to mild cases. The prominent lymphopenia, indicating an impairment of immune system, develops in most COVID-19 patients, especially in severe ones [3, 27]. Therefore, it seems that neutrophils and leukocytes might reinforce the CS rather than lymphocytes in COVID-19. 

The level of lymphocytes and subsets of T cells, which play a significant role in the balancing of the immune response, varies according to the type of the virus due to possible viral pathogenetic mechanisms. Data from recent studies have suggested that SARS-CoV-2 infection can lead to immune dysregulation by affecting the subsets of T cells. In patients with COVID-19, the levels of T helper cells (CD3+, CD4+), cytotoxic suppressor T cells (CD3+, CD8+), and regulatory T cells are below normal levels while T helper cells and T regulatory cells in severe patients are remarkably lower than nonsevere patients. Regulatory T cells are responsible for the maintenance of the immune homeostasis by suppressing the activation, proliferation, and proinflammatory function of most lymphocytes, including CD+4 T cells, CD+8 T cells, NK cells, and B cells [34, 35]. Furthermore, the percentage of naïve helper T cells amplifies, while the percentage of memory T helper cells and CD28+ cytotoxic suppressor T cells decreases in severe COVID-19 [3, 27]. The equilibrium between the naïve T cells and memory T cells is fundamental for maintaining an efficient immune response [36]. In addition to T cells, the reduction of B cells and NK cells are seen in COVID-19. Another important result is the confirmed strong relationship between inflammatory markers, including erythrocyte sedimentation rate (ESR), C-reactive protein (CRP) and IL-6, and the subset of lymphocytes [30]. However, there is no significant correlation between IL-6 with subsets of lymphocytes [3] and CD+4/CD+8 T cell ratio in SARS-CoV-2 infection is similar to the healthy group, the increase in this ratio and the decline of CD+8 T cells and B cells are considered poor predictors for the assessment of post-treatment clinical follow-up [3, 30]. Taken together, these results indicate that SARS-CoV-2 is responsible for an immune dysregulation with the induction of aberrant cytokine and chemokine response, alteration in levels of the lymphocyte subsets, all of which result in cytokine storm and sharpish tissue damage. 

## 4. Antiinflammatory treatments used for combatting COVID-19 

CS is an acute life-threatening dynamic inflammatory condition resulting from chaotic activation of a wide array of cytokines from initiation, immune hyperactivation, and eventually progressive multiorgan dysfunction/failure (MODS/MOF). Prompt diagnosis and effective therapeutic interventions are crucial for halting CS. Treatment is directed at controlling chaotic inflammatory response with specifically or nonspecifically targeting inflammatory cytokines or associated effector signaling pathways for restoring the host immune system (Table). Herein, we reviewed the role of the potential immuno- suppressive and immuno-modulatory treatments currently used in rheumatology practice to control hyper-inflammation encountered in COVID-19.

**Table:** Anti-rheumatic drugs with proven efficacy in the treatment of COVID-19.

Drug	Regimen	When to consider	Recommendations/precautions
Corticosteroids	For 10 days (or until discharge, if earlier)with daily doses of· Dexamethasone 6 mg IV or PO or · Prednisone 40 mg or· Methylprednisolone 32 mg Alternatively, 250 to 500 mg pulses for 3 consecutive days	Hospitalized patients with severe COVID-19 who require respiratory support (SpO2 ≤94% on room air) or with end organ dysfunction as seen in sepsis	· Concomitant use of antivirals is recommended· Beware of secondary viral, bacterial, fungal (i.e. Mucor mycosis) infections· Beware of reactivation of latent viral (i.e. Hepatitis B) and mycobacterial infections· Blood glucose monitoring· Steroid myopathy
Colchicine	0.5 mg PO BID for the first 3 days, and then once per day for 27 consecutive days	PCR positive outpatients with at least one adverse prognostic factor	· Evidence is weak for the use of colchicine· Bone marrow suppression, liver toxicity, diarrhea, myopathy, and serious drug interactions may occur with Cyp3A4 metabolites.
Anakinra	Not well determined, varied between 100 mg daily subc to 5 mg/kg twice a day IV. Doses used to treat HLH would be a reasonable approach(doses >4 mg per kg of body weight)	· Patients with obvious hyperinflammation (CRP >100 mg/L) who could not tolerate corticosteroids such as uncontrolled diabetes and with liver disease,· Refractory MIS-C and MIS-A	· Evidence is weak for the use of anakinra· Low subcutaneous doses (less than 200 mg/day) might not be effective· Not effective in advanced disease, ie in those who need ventilatory support on admission · More effective if used early before organ dysfunction· Not effective when co-administered with dexamethasone· Risk of neutropenia
Tocilizumab	400 mg iv single infusion, in case of respiratory decline a repeat dose of 400 mg 12–24 h after the first infusion	On top of corticosteroids when hypoxemia progressively worsens	· More effective if administered promptly at the time of rapidly progressive severe hypoxemia · Benefit might be unlikely if patients have received ventilatory support for several days or more· Beware of secondary infections and bowel perforation
Baricitinib	4 mg for 14 days or until hospital discharge	· Hospitalized patients requiring respiratory support· Should not be used for whom do not require oxygen support and for the COVID-19 prophylaxis	· 2 mg once daily for patients with estimated glomerular filtration rate of less than 60 ml/min · Greater efficacy if combined with corticosteroids · Beware of increased risk of thromboembolic events· Beware of secondary infections, particularly in patients who co-administered corticosteroids · Might increase the risk of a worse outcome if used for prophylaxis
Intravenous immuno-globulin (IVIg)	· 1–2 g per kg of body weight for up to 4 days, · MIS-C 2 g/kg single day along with pulse methyl prednisolone	· MIS-C & MIS-A patients · Refractory patients that meet WHO definition of critical COVID-19	· Low quality of evidence for its efficacy· Limited drug supply and extensive cost limits its use· Cardiac function and fluid status should be assessed before infusion. If abnormal, the rate of IVIg infusion may be slowed, or the treatment may be given in divided doses over 2 days, and/or diuretics may be considered to avoid volume overload · Increased risk of hemolytic anemia· A second dose of IVIg is not recommended in patients with refractory MIS-C

### 4.1. Corticosteroids

Initial case-based observations and, thereafter, the report of COVID-19 Global Rheumatology Alliance (GRA), which showed adverse outcomes in those patients receiving corticosteroids, precluded use of these drugs which is deemed to accelerate viral replication [37]. However, with a better understanding of the pathogenesis of severe COVID-19, corticosteroids are employed for their broad-spectrum antiinflammatory actions on production of proinflammatory cytokines and activation of immune cells, including T cells, monocytes and macrophages. Several well-designed clinical trials investigated the role of corticosteroids on various stages of the disease with different application regimens.

The largest corticosteroid trial is the open-label RECOVERY trial in which hospitalized COVID-19 patients were randomized to receive either standard of care (SOC, n=4321) or 6 mg/day dexamethasone (n=2104) for up to 10 days [38]. Dexamethasone reduced 28-day mortality rate by 20% (age-adjusted rate ratio 0.8, 95% CI 0.75–0.93) in patients requiring respiratory support as nasal, high-flow oxygen or invasive/noninvasive mechanical ventilation. However, a trend towards harm was noted with the use of dexamethasone in those patients who did not need respiratory support at the time of randomization. Therefore, guidelines https://www.idsociety.org/covid-19-real-time-learning-network/therapeutics-and-interventions/corticosteroids/ do not recommend the use of corticosteroids in patients with nonsevere COVID-19 (SpO2 >94%, not requiring supplemental oxygen). Studies regarding the effects of corticosteroids on viral clearance are conflicting, but there is a possibility that early administration of corticosteroids in nonseverely ill patients might be deleterious due to an increase of viral shedding or a delay in viral clearance [39, 40]. There is also evidence showing that impaired viral clearance is associated with severe disease and increased age rather than early use of corticosteroids [40]. 

Efficacy of intravenous dexamethasone was also investigated in COVID-19 patients with moderate to severe ARDS who required ICU admission. The CoDEX trial randomly allocated patients to receive either SOC alone (n=148) or 20 mg daily dexamethasone intravenously for 5 days, 10 mg of dexamethasone daily for 5 days or until ICU discharge plus SOC (n=151)[3, 41]. Dexamethasone provided more ventilator-free days during the first 28 days (6.6; 95% CI, 5.0-8.2 days vs. 4.0; 95% CI, 2.9–5.4 days; difference 2.26; 95% CI, 0.2–4.38 days) compared with SOC. However, all-cause mortality at 28 days was found similar between groups (56.3% in the dexamethasone group vs 61.5% the SOC group; hazard ratio, 0.97; 95% CI, 0.72–1.31). Dexamethasone was well-tolerated without increasing the rate of serious infections or hyperglycemia [3, 41]. 

Despite there were numerous clinical trials and several meta-analyses, the optimal corticosteroid type, dose, and treatment duration in patients with COVID-19 remains unclear. Dexamethasone is preferred over other corticosteroids for its shown efficacy in HLH but there are several other trials reporting positive results with the use of other agents like methylprednisolone in equivalent total daily doses of dexamethasone 6 mg/day (methylprednisolone 32 mg daily or prednisone 40 mg daily) [42–44]. Methylprednisolone 250 to 500 mg daily pulses for 3–6 consecutive days has also been shown to improve survival in severe COVID-19 patients [45, 46]. Hydrocortisone would be one of the options although its efficacy seemed to be lower than dexamethasone[46]. The open-label STOIC trial showed that, an inhaled steroid, budesonide 400 μg twice a day until symptom resolution, decreased the need for urgent medical care in mild patients with early COVID-19 [47].

Another possible role of corticosteroids would be the treatment of post-COVID inflammatory lung diseases, as suggested by preliminary data from small observational studies that report symptomatic and radiological improvement in a subset of patients with organizing pneumonia and lung fibrosis [48]. 

### 4.2. Chloroquine and hydroxychloroquine 

Chloroquine (CQ) and hydroxychloroquine (HCQ) are 4-aminoquinoline derivatives that are approved by the U.S. Food and Drug Administration (FDA) for the treatment of systemic lupus erythematosus, rheumatoid arthritis (RA), and they have been used for these disorders for decades. HCQ does not increase the risk of infection and has lipid-lowering, antithrombotic and antineoplastic properties [49]. CQ and HCQ may prolong QT interval constituting potential risk of fatal arrhythmia in higher doses or if combined with QT prolonging medications as well as those with cardiac diseases [50]. 

It has been known that CQ and HCQ have nonspecific antimicrobial activity against *Coxiella burnetii*, plasmodium, and many viruses, including hepatitis B, HIV, H1N1, and Zika virus [51]. Therefore, it had rapidly attracted attention at the beginning of COVID-19 pandemic. However, several randomized controlled trials clearly demonstrated that HCQ neither protected nor improved the outcomes of COVID-19 patients [52], leaving some important messages for the future pandemics. First, some trials showed a trend towards harm with use of HCQ [53]. Second, massive demand for HCQ caused drug shortage leading to the flare of disease in patients with lupus. Third, in-vitro studies and animal models do not necessarily mean in vivo efficacy of drugs when used for unapproved indications. 

### 4.3. Intravenous immunoglobulin (IVIg) 

IVIg is a blood product containing polyclonal immunoglobulin G isolated and pooled from healthy donors used to treat Immune Thrombocytopenic Purpura (ITP), Kawasaki disease and various inflammatory neurologic and myositis syndromes. It has immunomodulatory functions with unknown mechanisms of action. One of the proposed mechanisms is the interaction of IgG-Fc with Fc gamma receptors located on almost all immune cells, resulting in pleiotropic functional consequences including the expansion of regulatory T cell population, phagocytosis, antibody-dependent cellular cytotoxicity (ADCC), immune cell differentiation and maturation, apoptosis, expression of pro-inflammatory cytokines, chemokines, and antigen-presentation [54, 55]. 

Studies on efficacy of IVIg are largely retrospective cohort studies, and IVIg was utilized as a rescue medication following other medications like corticosteroids and tocilizumab [56, 57]. Prospective RCTs are scarce and included a small number of patients with conflicting results [56, 58, 59]. Metaanalyzes of published studies suggest survival advantage in only critical COVID-19 patients characterized by ARDS, sepsis, septic shock that require intensive care admission [60]. Moreover, its costliness and limited supply restrict its general use. The methodological limitations of all the studies preclude the extraction of any definitive conclusion about when and how to use IVIg in the context of COVID-19. However, patients with transplantation, pregnancy, secondary infections, immune thrombocytopenia, muscular, myocardial, and neurologic manifestations would be suitable candidates for IVIg treatment. It should be remembered that the efficacy of IVIg comes from immunomodulatory actions rather than viral neutralizing actions [61] which can be obtained at higher doses than neutralizing doses such as 1 g per kg body weight daily for up to 4 consecutive days [62]. 

American College of Rheumatology recommends the use of IVIg as the first-line treatment in patients complicated with Multisystem Inflammatory Syndrome in Children and Adults (MIS-C and MIS-A). In these patients IVIg is given 2 gr of per kg body weight for a single dose along with 1–2 mg/kg methylprednisolone, aspirin, and anti-coagulants [63–66].

### 4.4. IL-6 antagonists 

IL-6 receptors are expressed in almost all immune cells, and IL-6 acts as a master player inducing proliferation and differentiation of immune cells. IL-6 exerts pleiotropic effects on immune cells, which are manifested as promoted differentiation of T-helper type 17 (Th17), CD8+ T, and B cells, increased migration of neutrophils, and reduced development of Tregs. Moreover, IL-6 modulates endothelial cells and vascular smooth muscle cells leading to increased vascular permeability and leakage, endothelial activation, and accelerated atherogenesis, which are manifested clinically as cardiovascular events, hypotension, and pulmonary dysfunction in COVID-19. Collectively, IL-6 promotes both immune cell hyperactivation and target organ dysfunction in severe COVID-19 [67].

In healthy individuals, the IL-6 levels in circulation are extremely low and are in the range of 1–5 pg/mL, marked elevations reported in many inflammatory conditions including cytokine release syndrome [68]. Several therapeutic agents have been developed to inhibit the cytokine itself (sirikumab, siltuximab, clazakizumab, and olokizumab), the signaling via the IL-6 receptor (tocilizumab, sarilumab, levilimab), or its postreceptor downstream signaling pathways (JAK/STAT). Tocilizumab (TCZ) is approved for the treatment of rheumatoid arthritis (RA), juvenile idiopathic arthritis, giant cell arteritis, cytokine release syndrome, idiopathic multicentric Castleman’s disease (iMCD) FDA. Actemra (tocilizumab) injection, for intravenous or subcutaneous use: highlights of prescribing information.2010.www.accessdata.fda.gov/drugsatfda_docs/label/2017/125276s114lbl.pdf. 

COVID-19 patients have high plasma IL-6 levels, especially those with a more severe disease presentation [27]. IL-6 production can be stimulated by SARS-CoV-2 itself or by stimulation of other immune cells [69]. Indeed, it has been shown that, during COVID-19, CD4^+^T lymphocytes are rapidly activated to differentiate into pathogenic Th1 cells, generating GM-CSF and other pro-inflammatory cytokines, which further induce activation of monocytes with high expression of IL-6 [70]. In clinical point of view, there is striking correlation between serum IL-6 levels and SARS-CoV-2 RNAaemia, which strongly indicates worse outcome [71]. Hence, blocking IL-6 would potentially reduce the detrimental immune response caused by SARS-CoV-2. There are dozens of clinical trials assessing the efficacy of IL-6 antagonists in patients hospitalized for COVID-19 that variously reported benefit, no effect or harm, probably due to different study designs and enrolled patient characteristics. 

A recent metaanalysis evaluated 27 clinical trials performed on almost 11,000 patients with diverse disease characteristics at different dosages of IL-6 antagonists, either alone or combined with corticosteroids [72]. IL-6 antagonist use, compared to usual care or placebo, was associated with lower 28-day all-cause mortality, lower progression to invasive mechanical ventilation (IMV), or death without increasing the risk of infection at the same time period. Concurrent administration of IL-6 antagonists and corticosteroids at randomization provided greater benefit than either drug given individually. The association of IL-6 antagonists with lower mortality was more marked in those patients requiring oxygen flow rate of ≤15 L/min and noninvasive ventilation and who did not receive IMV at randomization (70). 

TCZ is the most widely studied IL-6 antagonist for the treatment of severe COVID-19, and its efficacy seemed to be more prominent than sarilimumab, while data are limited for other IL-6 antagonists [72]. Similarly, due to limited data, associations could not be determined for the efficacy of low-dose IL-6 antagonists, those who did not require respiratory support, use of concomitant antiviral treatment, and the risk of secondary infections in advance. Hence, it is prudent to monitor patients for the incidence of secondary/opportunistic infections and the other well-known complications such as bowel perforation. After accumulated data, FDA issued an emergency use authorization for the tocilizumab for the treatment of hospitalized adults and pediatric (≥2 years of age) COVID-19 patients who are requiring respiratory support and receiving systemic corticosteroids in June 2021. 

### 4.5. Janus kinase (JAK) inhibitors

JAK inhibitors are potent inhibitors of one or more of the JAK family of enzymes (JAK1, JAK2, JAK3, TYK2), interfering with the JAK-STAT signaling pathway, which mediates the effects of many interleukins (IL-2, IL-3, IL-4, IL-5, IL-6, IL-7, IL-9, IL-10, IL-12, IL-15, IL-21, IL-23), IFN-(α, β, γ) and growth factors (GM-CSF, TGF-β, erythropoietin and thrombopoietin) [73]. Like the other respiratory viruses, SARS-CoV-2 triggers inflammation via the JAK/STAT pathway leading to the recruitment of inflammatory cells, including macrophages, monocytes, lymphocytes, natural killer cells, and dendritic cells, as well as activation of pneumocytes and endothelial cells [74]. 

JAK inhibitors are currently approved for the treatment of RA, psoriatic arthritis, ulcerative colitis, polyarticular juvenile arthritis, and their use in other inflammatory disorders are continuously expanding [75]. Idea in COVID-19 treatment is that many pro-inflammatory cytokines involved in cytokine storm of COVID-19 might be inhibited by a single agent, JAK inhibitors (tofacitinib, baricitinib, ruxolitinib). Besides, these shared properties of JAK inhibitors, baricitinib blocks host Numb-associated kinases (AP-2-associated protein kinase 1, AAK1; cyclin G-associated kinase, GAK; BMP2 inducible kinase, BIKE), which all mediate viral entry [76]. This effect is only restricted to baricitinib among other JAK inhibitors with theoretic potential of blocking viral entry and assembly of virus particles into pneumocytes in therapeutic doses used to treat RA [77]. Given this hypothesis, baricitinib was tested in a large multi-center RCT, the Adaptive COVID-19 Treatment Trial 2 (ACTT-2)[78]. In this trial, a combination of remdesivir with baricitinib (4 mg/day for 14 days or until hospital discharge) showed improved outcomes (days for recovery, clinical status at day 15, and the 28-day mortality) compared to remdesivir used alone in hospitalized COVID-19 patients [78]. These clinical benefits were consistent across different age groups, sexes, and ethnic groups, as well as independent of the duration of symptoms and severity of disease at enrollment, though benefits of baricitinib combination were greater in moderate to severe COVID-19 patients requiring respiratory support as nasal, high flow oxygen or noninvasive ventilation. Although combination did not reveal a significant 28-day mortality difference (5.1% with remdesivir plus baricitinib vs. 7.8% with remdesivir alone), the composite outcome progression to mechanical ventilation and death was prevented in one third of patients (HR 0.69; 95% CI, 0.50–0.95). Moreover, remdesivir plus baricitinib reduced the mechanical ventilation or extracorporeal membrane oxygenation (ECMO) requirements by median of 11 days among patients who required these interventions after enrollment [78]. With the results of this trial, FDA issued an emergency use authorization for remdesivir plus baricitinib in hospitalized patients with COVID-19 who required oxygen supplementation in November 2020. 

COV-BARRIER study investigated the role of baricitinib on a heterogenous group of patients [79]. Patients were randomly assigned to receive either baricitinib 4 mg 14 days (n=764) or placebo (n=761) on top of standard of care determined by local clinical practice. Only %20 of patients co-administered remdesivir and more than 90% of patients received concomitant dexamethasone. Although baricitinib did not reach the composite primary endpoint (the proportion of patients who progressed to high-flow oxygen, noninvasive ventilation, invasive mechanical ventilation, or death by day 28), the 28-day all-cause mortality was 8% for baricitinib and 13% for placebo (HR 0.57; 95% CI, 0.41–0.78) revealing a 38% relative reduction in mortality. Consistently, the 60-day all-cause mortality was 10% for baricitinib vs 15% for placebo (HR 0.62; 95%CI, 0.47–0.83). According to intention to treat analysis, one additional death was prevented per 20 baricitinib-treated patients, a number like ACTT-2 trial. Benefits were consistent across different age groups, sexes, and ethnic groups, duration of symptoms and baseline corticosteroid use. Observed benefits were greater in patients with severe disease and who did not receive remdesivir at baseline. After the result of this trial, FDA has revised the Emergency Use Authorization, authorizing baricitinib alone for the treatment of COVID-19 in hospitalized adults and pediatric patients (≥2 years of age), requiring respiratory support or ECMO in July 2021 [79]. 

Efficacy of another JAK inhibitor, tofacitinib in COVID-19 pneumonia was investigated an industry-supported trial in 289 patients in Brazil (STOP-COVID)[80]. Tofacitinib was administered 10 mg twice daily for up to 14 days or until hospital discharge to patients requiring respiratory support. Although the STOP-COVID trial was not powered to detect a difference in mortality, it has reached the composite primary endpoint, which was the reduction of death or respiratory failure at 28 days (18.1% of the tofacitinib vs 29.0% of the placebo, risk ratio 0.63; 95% CI, 0.41–0.97). Of note, while 20% of the patients were co-administered corticosteroids in the ACTT-2 trial, almost 90% of patients received them in STOP-COVID trial, suggesting the superiority of combination. Studies for another JAK inhibitor, ruxolitinib, are ongoing and preliminary results are promising [81].

JAK inhibitors increase the risk of secondary infections and thromboembolic events, particularly in patients with underlying cardiovascular disease. However, compared to placebo, baricitinib and tofacitinib had a significantly lower incidence of severe/nonsevere adverse events including infections [78, 80], though patients who received concomitant glucocorticoids after randomization had a higher rate of incident infections [78]. COVID-19 Global Rheumatology Alliance reported a two-fold increased risk of mechanical ventilation and death in RA patients who were under treatment with JAK inhibitors compared to TNF inhibitors[82]. In the same study, investigators also find more than 4-fold increased risk of worse outcomes for rituximab, whereas IL-6 inhibitors and abatacept did not increase adverse outcomes compared to anti-TNF users [78]. Hence, timing of JAK-inhibitor initiation during COVID-19 might have pivotal importance in determining the outcome. During initial phases of SARS-CoV-2 infection, symptomatic patients present with impaired type I/III IFN-mediated antiviral responses. Critically ill COVID-19 patients show genetic polymorphisms in one IFN receptor gene (IFNRA2) and in a gene locus near the TYK2, which is the key for IFN, interleukin (IL)-12 and IL-23 signaling, and Th1/Th17 cell-mediated antiviral immune responses [83]. Pretreatment with JAK inhibitors might blunt IFNs’ antiviral responses increasing risk of severe infection; therefore, these group of drugs should not be used for prophylaxis or those not requiring respiratory support [83]. In patients with moderate to severe SARS-CoV-2 pneumonia, combination of baricitinib with corticosteroids provided greater improvement in pulmonary functions than corticosteroids alone; therefore, it would be better to co-administer JAK inhibitors with corticosteroids [80, 84].

### 4.6. IL-1 inhibitors: anakinra and canakinumab

IL-1 family are pleiotropic cytokines, have roles in inflammation, hematopoiesis, and fibrosis. IL-1β and TNF-α promote vascular permeability and leakage. Both IL-1β and IL-18 fuel cytokine storm and MAS and IL-1 cytokines (except IL-18) can be successfully inhibited by IL-1 antagonists [85]. Nod-like receptor family pyrin domain-containing 3 (NLRP3) is a critical inflammasome in the acute protection of the body against a wide variety of noxious stimuli, including RNA viruses [86]. SARS-COV-2 has been shown to induce NLRP3 by its ion channel-forming M protein and ORF8b[87] and activates caspase-1, a molecule responsible for the activation and exuberant release of IL-1β and IL-18 [26, 27, 88].

Anakinra FDA. Kineret® (anakinra) for injection, for subcutaneous use: Highlights Of prescribing Information. 2001. https://www.accessdata.fda.gov/drugsatfda_docs/label/2012/103950s5136lbl.pdf is a recombinant antagonist of human IL-1 and approved for the treatment of RA and certain autoinflammatory disorders with recommended doses of 1–2 mg/kg/day with a maximum daily dose of 8 mg/kg. In terms of sepsis and MAS, the use of extremely high doses of anakinra (2 mg/kg/h for 72 h continuous infusion) was shown to be safe [89]. Because of its proven safety, anakinra is one of the first studied immunomodulatory treatments showing benefit when it’s used at higher doses but not with low doses [90]. Currently, there are several anakinra studies registered for COVID-19 and only one RCT; the CORIMUNO-ANA-1 trial has published results [91]. The rest of the available data come from low-quality studies with a significant risk of bias. The CORIMUNO-ANA-1 trial included mild-to-moderate COVID-19 inpatients who required at least 3 L/min of oxygen but not mechanical ventilation and elevated CRP (≥25 mg/L). Patients (n=116) were 1:1 randomized to receive either SOC or SOC plus intravenous anakinra (200 mg twice a day on days 1–3, 100 mg twice on day 4, and 100 mg once on day 5, a similar dose used to treat critically ill patients with HLH. The trial was early terminated following an interim analysis that showed any evidence to support anakinra on clinical improvement, ventilator support and death over SOC alone. However, this trial is criticized for use of corticosteroids in more than half of patients in both arms that might shadow efficacy of anakinra. A recent metaanalysis revealed improved survival in patients treated with anakinra (38 deaths in 342) than in those who received SOC (137 deaths in 553; adjusted OR 0.32 95%CI, 0.20–0.51) [92]. Subgroup analyzes showed that anakinra was more effective in reducing mortality in patients with CRP concentrations of >100 mg/L and when given without dexamethasone, but it was not effective when co-administered with dexamethasone. Metaanalysis also reported a nonsignificant increase in the risk of adverse events with anakinra [92]. The SAVE study determined high-risk patients, which potentially progress into severe respiratory failure by measuring serum soluble urokinase plasminogen activator receptor (suPAR) levels as an early indicator for clinical worsening. Patients were treated with 100 mg/day subcutaneous anakinra for 10 days if they had suPAR of >6 ng/mL reporting improved respiratory and survival outcomes suggesting superior efficacy when administered early [93, 94]. The other IL-1β antagonist canakinumab (450–750 mg single iv infusion) was failed to show efficacy in improving survival without IMV [95].

### 4.7. Colchicine 

Colchicine has multiple effects on the function of the immune system, particularly on neutrophils, including inhibition of tubulin polymerization and microtubule generation, chemotaxis, superoxide anion production, suppression of cellular adhesion molecules, inflammatory chemokines, and cytokines (TNF-α and IL-6) as well as NLRP3 inflammasome activation that mediates interleukin 1β production [96]. Colchicine is approved for the treatment of gout and familial Mediterranean fever (FMF) but has attracted attention in recent years for the management of atherosclerotic cardiovascular disease and idiopathic pericarditis [97]. Beyond gout and FMF, given its anti-inflammatory/immuno-modulatory properties and good safety profile, colchicine is widely used to treat a variety of inflammatory conditions. Several studies investigated the efficacy of colchicine in COVID-19, yielding conflicting results. 

The first small RCT (n=36 in both arms) by Lopes et al, suggested superiority of colchicine on reduction in length of both supplemental oxygen therapy and hospitalization compared to placebo in patients with moderate to severe COVID-19 but fail to show survival benefit due to the low number of deaths in both groups [98]. The COLCORONA trial included outpatients, diagnosed within 24 h of randomization, over 40 years with at least one high-risk adverse prognostic feature to determine the composite endpoint of death or hospital admission. Patients were 1:1 randomized to receive either colchicine (n=2235) 0.5 mg twice per day for 3 days followed by once per day for 27 more days or placebo (n=2253) [99]. The primary endpoint evolved in 104 (4.7%) in the colchicine group and 131 (5.8%) in the placebo group (OR: 0.79, 95% CI, 0.61-1.03; p=0.08), reaching significance when only PCR confirmed 4159 cases were included (OR 0.75, 95%CI, 0.57–0.99; p=0.042). Death was occurred in 5 (0.2%) and 9 (0.4%) patients (OR 0.56, 95%CI, 0.19–1.66) in colchicine and placebo groups, respectively. In RECOVERY, colchicine arm of the trial was terminated, since the colchicine failed to show a significant difference in the primary endpoint of the 28-day mortality rate of colchicine vs. SOC alone (21% in both colchicine and SOC arms; risk ratio 1.01, 95%CI: 0.93-1.1; P=0.77) [100]. 

Metaanalyzes of observational and RCTs showed an advantage of colchicine use; however, subgroup analysis with randomized controlled trials showed no statistically significant difference in the mortality (OR: 0.80, 95%CI, 0.44–1.46) [101]. Moreover, colchicine neither prevented COVID-19-related hospital admissions nor disease course among rheumatic patients who were receiving it for their underlying diseases [102, 103]. Colchicine has potential toxicities, particularly in those with kidney and liver impairment and elders, myelosuppression, elevation in transaminases, and myopathy, which are commonly observed during the course of COVID-19 [97, 104]. Therefore, routine use of colchicine is not recommended until well designed trials show its clear benefit on specific populations.

### 4.8. Anti-TNF agents

TNF-α is one of the most potent pro-inflammatory cytokines with a broad spectrum of actions. TNF-α is produced by macrophages, monocytes, and T cells that promote expression or other inflammatory cytokines via NFKB pathway. Marked elevations were reported in many inflammatory conditions including cytokine release syndrome. [26]. SARS-CoV viral spike protein can modulate TNF-α-converting enzyme (TACE)-dependent shedding of the ACE2 ectodomain, required for the viral entry which is coupled to TNF-α production [105]. However, studies assessing serum TNF-α levels in COVID-19 patients revealed conflicting results. Some studies found that it elevated, whereas some others did not [67]. High serum TNF concentrations at the time of admission, thus, at very early phase of infection, predicted poor outcomes and found significantly elevated in severe COVID patients [106]. Therefore, it’s hypothesized that the use of TNF inhibitors might be effective in blocking viral entry and detrimental effects of exuberant TNF-α, as shown in preclinical studies on severe respiratory syncytial virus and influenza infections [106, 107]. The COVID-19 Global Rheumatology Alliance (GRA) registry showed fewer hospitalizations among people treated with TNF antagonists than non-TNF inhibitor biologic/JAK inhibitor users (adjusted OR 0.40, 95%CI, 0.19–0.81) that indicate the potential favorable role of anti-TNFs [82]. 

Since TNF fuels other potent pro-inflammatory cytokines, anti-TNF therapy might be more effective when applied early in the disease course to block excessive production of TNF. Intravenous infliximab and subcutaneous adalimumab with loading doses might be preferred over other anti-TNFs. Although there are few observational reports regarding the successful use of anti-TNFs, the limitations of observational data need to be considered when translating these findings to clinical practice. Trials on the use of anti-TNFs in COVID-19 such as the “adalimumab in COVID-19 to prevent respiratory failure in community care, (AVID-CC)” are ongoing, but none of them have published results. 

### 4.9. Anti-IL-17 antagonists 

One of the cytokines found abundant in COVID-19 patients is IL-17 and reported to be associated with severe lung inflammation [26]. IL-17 has wide-ranging pro-inflammatory effects on induction of cytokines; IL-1β, IL-6, TNF-α; growth factors, G-CSF; chemokines; and matrix metalloproteinases. IL-17 inhibitors did not increase the risk of progressive COVID-19 [108]. For the use of IL-17 antagonists in COVID-19, there are very scarce data, limited to a recent study from Russia, which fail to show clinical benefit of netakimab [109].

### 4.10. GM-CSF inhibitors 

GM-CSF is one of the key molecules involved in cytokine storm which is excessively released in COVID-19 patients [70]. GM-CSF is crucial for driving both innate and adaptive immune responses and blockade of this growth factor may halt immunopathology caused by the virus. Mavrilimumab is a GM-CSF inhibitor developed for the refractory RA [110]. Studies evaluating the efficacy of mavrilimumab revealed conflicting results [41, 111]. Lenzilumab is another GM-CSF inhibitor, which show promise in the treatment of severe refractory COVID-19 [112]. 

## 5. Conclusion

Excessive inflammatory response with features of cytokine storm causes severe disease course and adversely affects the outcome of COVID-19. Successful vaccination campaigns and antiviral drugs in development programs would be the most effective tools in the fight against the COVID-19. Larger trials are ongoing, and their results are urgently needed to ascertain the most effective treatment options, and some of such novel agents show promise in their early clinical studies. Until the discovery of curative medications, drugs that are used in daily rheumatology practice constitute life-saving treatment options in COVID-19 patients with an extenuating severe detrimental inflammatory response. Herein, we discussed altered immune responses in COVID-19 and presented current treatment options in the treatment of severe COVID-19.

## References

[ref1] Kuri-Cervantes L Pampena MB Meng W Rosenfeld AM Ittner CAG 2020 Comprehensive mapping of immune perturbations associated with severe COVID-19 5 10.1126/sciimmunol.abd7114 PMC7402634 32669287

[ref2] Xu Z Shi L Wang Y Zhang J Huang L 2020 Pathological findings of COVID-19 associated with acute respiratory distress syndrome The Lancet Respiratory Medicine 10 30076 x 10.1016/S2213-2600(20)30076-X PMC7164771 32085846

[ref3] Tomazini BM Maia IS Cavalcanti AB Berwanger O Rosa RG 2020 Effect of Dexamethasone on Days Alive and Ventilator-Free in Patients With Moderate or Severe Acute Respiratory Distress Syndrome and COVID-19: The CoDEX Randomized Clinical Trial Journal of the American Medical Association 324 1307 1316 32876695 10.1001/jama.2020.17021 PMC7489411

[ref4] Guan WJ Ni ZY Hu Y Liang WH Ou CQ 2020 Clinical Characteristics of Coronavirus Disease 2019 in China. New England Journal of Medicine 382 1708 1720 10.1056/NEJMoa2002032 PMC7092819 32109013

[ref5] Gustine JN Jones D 2021 Immunopathology of Hyperinflammation in COVID-19. American Journal of Pathology 191 4 17 10.1016/j.ajpath.2020.08.009 PMC7484812 32919977

[ref6] Schultze JL Aschenbrenner AC 2021 COVID-19 and the human innate immune system Cell 184 1671 1692 33743212 10.1016/j.cell.2021.02.029 PMC7885626

[ref7] Yang Y. Shen C. Li J. Yuan J. Wei J. 2020 Plasma IP-10 and MCP-3 levels are highly associated with disease severity and predict the progression of COVID-19 The Journal of allergy and clinical immunology 146 119 127 32360286 10.1016/j.jaci.2020.04.027 PMC7189843

[ref8] Ashour HM Elkhatib WF Rahman MM Elshabrawy HA 2020 Insights into the Recent 2019 Novel Coronavirus (SARS-CoV-2) in Light of Past Human Coronavirus Outbreaks 9 10.3390/pathogens9030186 PMC7157630 32143502

[ref9] Ziebuhr J Snijder EJ Gorbalenya AE 2000 Virus-encoded proteinases and proteolytic processing in the Nidovirales Journal of General Virology 81 853 879 10.1099/0022-1317-81-4-853 10725411

[ref10] Baez-Santos YM St John SE Mesecar AD 2015 The SARS-coronavirus papain-like protease: structure, function and inhibition by designed antiviral compounds Antiviral Research 115 21 38 25554382 10.1016/j.antiviral.2014.12.015 PMC5896749

[ref11] Lu X Pan J Tao J Guo D. 2011 SARS-CoV nucleocapsid protein antagonizes IFN-beta response by targeting initial step of IFN-beta induction pathway, and its C-terminal region is critical for the antagonism Virus Genes 42 37 45 20976535 10.1007/s11262-010-0544-x PMC7088804

[ref12] Ujike M 2015 Incorporation of spike and membrane glycoproteins into coronavirus virions Viruses 7 1700 1725 25855243 10.3390/v7041700 PMC4411675

[ref13] Masters PS 2006 The molecular biology of coronaviruses Advances in Virus Research 66 193 292 16877062 10.1016/S0065-3527(06)66005-3 PMC7112330

[ref14] Neuman BW Kiss G Kunding AH Bhella D Baksh MF 2011 A structural analysis of M protein in coronavirus assembly and morphology Journal of Structural Biology 174 11 22 21130884 10.1016/j.jsb.2010.11.021 PMC4486061

[ref15] Schoeman D Fielding BC 2019 Coronavirus envelope protein: current knowledge Virology Journal 16 69 69 31133031 10.1186/s12985-019-1182-0 PMC6537279

[ref16] Nieto-Torres JL DeDiego ML Verdiá-Báguena C Jimenez-Guardeño JM Regla-Nava JA 2014 Severe acute respiratory syndrome coronavirus envelope protein ion channel activity promotes virus fitness and pathogenesis PLoS Pathogens 10 10.1371/journal.ppat.1004077 PMC4006877 24788150

[ref17] Xia S Zhu Y Liu M Lan Q Xu W 2020 Fusion mechanism of 2019-nCoV and fusion inhibitors targeting HR1 domain in spike protein Cellular & Molecular Immunology 10 10.1038/s41423-020-0374-2 PMC7075278 32047258

[ref18] Zhou P Yang X-L Wang X-G Hu B Zhang L 2020 Discovery of a novel coronavirus associated with the recent pneumonia outbreak in humans and its potential bat origin bioRxiv 914952

[ref19] Hoffmann M Kleine-Weber H Schroeder S Kruger N Herrler T 2020 SARS-CoV-2 Cell Entry Depends on ACE2 and TMPRSS2 and Is Blocked by a Clinically Proven Protease Inhibitor Cell 10 10.1016/j.cell.2020.02.052 PMC7102627 32142651

[ref20] Ou X Liu Y Lei X Li P Mi D 2020 Characterization of spike glycoprotein of SARS-CoV-2 on virus entry and its immune cross-reactivity with SARS-CoV Nature Communications 11 1620 1620 10.1038/s41467-020-15562-9 PMC7100515 32221306

[ref21] Ivashkiv LB Donlin LT 2014 Regulation of type I interferon responses Nature Reviews Immunology 14 36 49 10.1038/nri3581 PMC4084561 24362405

[ref22] Li G Fan Y Lai Y Han T Li Z 2020 Coronavirus infections and immune responses Journal of Medical Virology 92 424 432 31981224 10.1002/jmv.25685 PMC7166547

[ref23] George MR 2014 Hemophagocytic lymphohistiocytosis: review of etiologies and management Journal of Blood Medicine 5 69 86 24966707 10.2147/JBM.S46255 PMC4062561

[ref24] Ramos-Casals M Brito-Zeron P Lopez-Guillermo A Khamashta MA Bosch X 2014 Adult haemophagocytic syndrome Lancet 383 1503 1516 24290661 10.1016/S0140-6736(13)61048-X

[ref25] McGonagle D Sharif K O’Regan A Bridgewood C 2020 Interleukin-6 use in COVID-19 pneumonia related macrophage activation syndrome Autoimmunity Reviews 102537 10.1016/j.autrev.2020.102537 PMC7195002 32251717

[ref26] Huang C Wang Y Li X Ren L Zhao J 2020 Clinical features of patients infected with 2019 novel coronavirus in Wuhan, China 395 497 506 10.1016/S0140-6736(20)30183-5 PMC7159299 31986264

[ref27] Qin C Zhou L Hu Z Zhang S Yang S 2020 Dysregulation of immune response in patients with COVID- China. Clinical Infectious Diseases 10 10.1093/cid/ciaa248 PMC7108125 32161940

[ref28] Liu Y Yang Y Zhang C Huang F Wang F 2020 Clinical and biochemical indexes from 2019-nCoV infected patients linked to viral loads and lung injury Science China Life Sciences 63 364 374 32048163 10.1007/s11427-020-1643-8 PMC7088566

[ref29] Özger HS Aysert Yıldız P Gaygısız Ü Uğraş Dikmen A Demirbaş Gülmez 2020 The factors predicting pneumonia in COVID-19 patients: preliminary results from a university hospital in Turkey Turkish Journal of Medical Sciences 50 1810 1816 32599972 10.3906/sag-2005-385 PMC7775687

[ref30] Wang F Nie J Wang H Zhao Q Xiong Y 2020 Characteristics of peripheral lymphocyte subset alteration in COVID-19 pneumonia Journal of Infectious Diseases 221 1762 1769 10.1093/infdis/jiaa150 PMC7184346 32227123

[ref31] Crayne CB Albeituni S Nichols KE Cron RQ 2019 The Immunology of Macrophage Activation Syndrome 10 119 119 10.3389/fimmu.2019.00119 PMC6367262 30774631

[ref32] Wang W Liu X Wu S Chen S Li Y 2020 Definition and Risks of Cytokine Release Syndrome in 11 Critically Ill COVID-19 Patients With Pneumonia: Analysis of Disease Characteristics Journal of infectious diseases 222 1444 1451 10.1093/infdis/jiaa387 PMC7337810 32601708

[ref33] Zinkernagel RM 1996 Immunology taught by viruses Science 271 173 178 8539616 10.1126/science.271.5246.173

[ref34] Sakaguchi S Yamaguchi T Nomura T Ono M. Regulatory 2008 T cells and immune tolerance Cell 133 775 787 18510923 10.1016/j.cell.2008.05.009

[ref35] Sakaguchi S Miyara M Costantino CM Hafler DA 2010 FOXP3+ regulatory T cells in the human immune system Nature Reviews Immunolgy 10 490 500 10.1038/nri2785 20559327

[ref36] Sallusto F Lenig D Forster R Lipp M Lanzavecchia A 1999 Two subsets of memory T lymphocytes with distinct homing potentials and effector functions Nature 401 708 712 10537110 10.1038/44385

[ref37] Gianfrancesco M Hyrich KL Al-Adely S Carmona L Danila MI 2020 Characteristics associated with hospitalisation for COVID-19 in people with rheumatic disease: data from the COVID-19 Global Rheumatology Alliance physician-reported registry Annals of Rheumatic Diseases 79 859 866 10.1136/annrheumdis-2020-217871 PMC7299648 32471903

[ref38] 2021 Dexamethasone in Hospitalized Patients with Covid-19. New England Journal of Medicine 384 693 704 10.1056/NEJMoa2021436 PMC7383595 32678530

[ref39] Romanou V Koukaki E Chantziara V Stamou P Kote A 2021 Dexamethasone in the Treatment of COVID-19: Primus Inter Pares? Journal of Personalized Medicine 11 10.3390/jpm11060556 PMC8232727 34203880

[ref40] Spagnuolo V Guffanti M Galli L Poli A Querini PR 2020 Viral clearance after early corticosteroid treatment in patients with moderate or severe covid-19 Scientific Reports 10 21291 21291 33277573 10.1038/s41598-020-78039-1 PMC7718220

[ref41] De Luca G Cavalli G Campochiaro C Della-Torre E Angelillo P 2020 GM-CSF blockade with mavrilimumab in severe COVID-19 pneumonia and systemic hyperinflammation: a single-centre, prospective cohort study Lancet Rheumatolgy 2 e465 e473 10.1016/S2665-9913(20)30170-3 PMC7430344 32835256

[ref42] Fatima SA Asif M Khan KA Siddique N Khan AZ 2020 Comparison of efficacy of dexamethasone and methylprednisolone in moderate to severe covid 19 disease Annals of Medicine and Surgery (Lond) 60 413 416 10.1016/j.amsu.2020.11.027 PMC7654232 33200031

[ref43] Du Plessis EM Lalla U Allwood BW Louw EH Nortje A 2021 Corticosteroids in critical COVID-19: are all corticosteroids equal? The South African Medical Journal 111 550 553 34382564

[ref44] Therapies 2020 WHO Rapid Evidence Appraisal for COVID- Journal of the American Medical Association 324 1330 1341 32876694

[ref45] Edalatifard M Akhtari M Salehi M Naderi Z Jamshidi A 2020 Intravenous methylprednisolone pulse as a treatment for hospitalised severe COVID-19 patients: results from a randomised controlled clinical trial European Respiratory Journal 56 02808 2020 10.1183/13993003.02808-2020 PMC7758541 32943404

[ref46] Cui Y Sun Y Sun J Liang H Ding X 2021 Efficacy and Safety of Corticosteroid Use in Coronavirus Disease 2019 (COVID-19): A Systematic Review and Meta-Analysis Infectious Diseases and Therapy 10 10.1007/s40121-021-00518-3 PMC8363240 34389970

[ref47] Ramakrishnan S Nicolau DV Jr. Langford B Mahdi M Jeffers H 2021 Inhaled budesonide in the treatment of early COVID-19 (STOIC): a phase 2, open-label, randomised controlled trial Lancet Respiratory Medicine 9 763 772 33844996 10.1016/S2213-2600(21)00160-0 PMC8040526

[ref48] Myall KJ Mukherjee B Castanheira AM Lam JL Benedetti G 2021 Persistent Post-COVID-19 Interstitial Lung Disease. An Observational Study of Corticosteroid Treatment. Annals of the American Thoracic Society 18 799 806 33433263 10.1513/AnnalsATS.202008-1002OC PMC8086530

[ref49] Al-Bari MA 2015 Chloroquine analogues in drug discovery: new directions of uses, mechanisms of actions and toxic manifestations from malaria to multifarious diseases Journal of Antimicrobial Chemotherapy 70 1608 1621 10.1093/jac/dkv018 PMC7537707 25693996

[ref50] Costedoat-Chalumeau N Hulot JS Amoura Z Leroux G Lechat P 2007 Heart conduction disorders related to antimalarials toxicity: an analysis of electrocardiograms in 85 patients treated with hydroxychloroquine for connective tissue diseases Rheumatology (Oxford) 46 808 810 17202178 10.1093/rheumatology/kel402

[ref51] ’Alessandro D Scaccabarozzi S Signorini D Perego L Ilboudo F DP 2020 The Use of Antimalarial Drugs against Viral Infection Microorganisms 8 10.3390/microorganisms8010085 PMC7022795 31936284

[ref52] 2020 Effect of Hydroxychloroquine in Hospitalized Patients with Covid-19. New England Journal of Medicine 383 2030 2040 10.1056/NEJMoa2022926 PMC7556338 33031652

[ref53] Axfors C Schmitt AM Janiaud P Hooft J Abd-Elsalam S 2021 Mortality outcomes with hydroxychloroquine and chloroquine in COVID-19 from an international collaborative meta-analysis of randomized trials Nature Communications 12 2349 2349 10.1038/s41467-021-22446-z PMC8050319 33859192

[ref54] Arumugham VB Rayi A. Intravenous 2020 Immunoglobulin (IVIG). StatPearls. Treasure Island (FL): StatPearls Publishing LLC 32119333

[ref55] Rodriguez de la Concepcion ML Ainsua-Enrich E Reynaga E Avila-Nieto C Santos JR 2021 High-dose intravenous immunoglobulins might modulate inflammation in COVID-19 patients 4 10.26508/lsa.202001009 PMC8321664 34321327

[ref56] Liu J Chen Y Li R Wu Z Xu Q 2021 Intravenous immunoglobulin treatment for patients with severe COVID-19: a retrospective multicentre study Clinical Microbiology and Infection 27 1488 1493 34020032 10.1016/j.cmi.2021.05.012 PMC8131555

[ref57] Omma A Erden A Armagan B Guven SC Karakas O 2021 A single center experience of intravenous immunoglobulin treatment in Covid-19 98 107891 107891 10.1016/j.intimp.2021.107891 PMC8200303 34153671

[ref58] Tabarsi P Barati S Jamaati H Haseli S Marjani M 2021 Evaluating the effects of Intravenous Immunoglobulin (IVIg) on the management of severe COVID-19 cases: A randomized controlled trial International Immunopharmacology 90 107205 107205 33214093 10.1016/j.intimp.2020.107205 PMC7665876

[ref59] Gharebaghi N Nejadrahim R Mousavi SJ Sadat-Ebrahimi SR Hajizadeh R. 2020 The use of intravenous immunoglobulin gamma for the treatment of severe coronavirus disease 2019: a randomized placebo-controlled double-blind clinical trial BMC Infectious Diseases 20 786 786 33087047 10.1186/s12879-020-05507-4 PMC7576972

[ref60] Xiang HR Cheng X Li Y Luo WW Zhang QZ 2021 Efficacy of IVIG (intravenous immunoglobulin) for corona virus disease 2019 (COVID-19): A meta-analysis International Immunopharmacology 96 107732 107732 34162133 10.1016/j.intimp.2021.107732 PMC8084608

[ref61] Kubota-Koketsu R Terada Y Yunoki M Sasaki T Nakayama EE 2021 Neutralizing and binding activities against SARS-CoV-1/2, MERS-CoV, and human coronaviruses 229E and OC43 by normal human intravenous immunoglobulin derived from healthy donors in Japan 61 356 360 10.1111/trf.16161 33104267

[ref62] Mascolo S Carleo MA Contieri M Izzo S Perna A 2021 SARS-CoV-2 and inflammatory responses: From mechanisms to the potential therapeutic use of intravenous immunoglobulin Journal of Medical Virology 93 2654 2661 33150961 10.1002/jmv.26651

[ref63] Henderson LA Canna SW Friedman KG Gorelik M Lapidus SK Rheumatology Arthritis 2021 American College of Rheumatology Clinical Guidance for Multisystem Inflammatory Syndrome in Children Associated With SARS-CoV-2 and Hyperinflammation in Pediatric COVID-19: Version 2 73 e13 e29 10.1002/art.41616 PMC8559788 33277976

[ref64] Ouldali N Toubiana J Antona D Javouhey E Madhi F 2021 Association of Intravenous Immunoglobulins Plus Methylprednisolone vs Immunoglobulins Alone With Course of Fever in Multisystem Inflammatory Syndrome in Children Journal of the American Medical Association 325 855 864 33523115 10.1001/jama.2021.0694 PMC7851757

[ref65] Emeksiz S Celikel Acar B Kibar AE Ozkaya Parlakay A Perk O 2021 Algorithm for the diagnosis and management of the multisystem inflammatory syndrome in children associated with COVID-19 International Journal of Clininical Practice 75 10.1111/ijcp.14471 PMC8237077 34107136

[ref66] Son MBF Murray N Friedman K Young CC Newhams MM 2021 Multisystem Inflammatory Syndrome in Children - Initial Therapy and Outcomes. New England Journal of Medicine 385 23 34 10.1056/NEJMoa2102605 PMC8220972 34133855

[ref67] Yang L Xie X Tu Z Fu J Xu D Therapy Targeted 2021 The signal pathways and treatment of cytokine storm in COVID-19 6 255 255 10.1038/s41392-021-00679-0 PMC8261820 34234112

[ref68] Uciechowski P Dempke WCM 2020 Interleukin-6: A Masterplayer in the Cytokine Network Oncology 98 131 137 31958792 10.1159/000505099

[ref69] Liao Y Wang X Huang M Tam JP Liu DX 2011 Regulation of the p38 mitogen-activated protein kinase and dual-specificity phosphatase 1 feedback loop modulates the induction of interleukin 6 and 8 in cells infected with coronavirus infectious bronchitis virus Virology 420 106 116 21959016 10.1016/j.virol.2011.09.003 PMC7111953

[ref70] Zhou Y Fu B Zheng X Wang D Zhao C Qi Y 2020 Pathogenic T-cells and inflammatory monocytes incite inflammatory storms in severe COVID-19 patients 7 998 1002 10.1093/nsr/nwaa041 PMC7108005 34676125

[ref71] Chen X Zhao B Qu Y Chen Y Xiong J 2020 Detectable Serum Severe Acute Respiratory Syndrome Coronavirus 2 Viral Load (RNAemia) Is Closely Correlated With Drastically Elevated Interleukin 6 Level in Critically Ill Patients With Coronavirus Disease 2019 Clinical Infectious Disease 71 1937 1942 10.1093/cid/ciaa449 PMC7184354 32301997

[ref72] Therapies 2021 WHO Rapid Evidence Appraisal for COVID- Journal of the American Medical Association 326 499 518 34228774

[ref73] A TV Haikarainen T Raivola J Silvennoinen O. Selective 2019 JAKinibs: Prospects in Inflammatory and Autoimmune Diseases BioDrugs 33 15 32 30701418 10.1007/s40259-019-00333-w PMC6373396

[ref74] Satarker S Tom AA Shaji RA Alosious A Luvis M 2021 JAK-STAT Pathway Inhibition and their Implications in COVID-19 Therapy. Postgraduate Medical Journal 133 489 507 10.1080/00325481.2020.1855921 PMC7784782 33245005

[ref75] Fragoulis GE McInnes IB Siebert S. 2019 JAK-inhibitors. New players in the field of immune-mediated diseases, beyond rheumatoid arthritis Rheumatology (Oxford) 58 i43 i54 30806709 10.1093/rheumatology/key276 PMC6390879

[ref76] Stebbing J Sanchez Nievas G Falcone M Youhanna S Richardson P 2021 JAK inhibition reduces SARS-CoV-2 liver infectivity and modulates inflammatory responses to reduce morbidity and mortality Science Advances 7 10.1126/sciadv.abe4724 PMC7775747 33187978

[ref77] Richardson P Griffin I Tucker C Smith D Oechsle O 2020 Baricitinib as potential treatment for 2019-nCoV acute respiratory disease Lancet 395 e30 e31 32032529 10.1016/S0140-6736(20)30304-4 PMC7137985

[ref78] Kalil AC Patterson TF Mehta AK Tomashek KM Wolfe CR 2021 Baricitinib plus Remdesivir for Hospitalized Adults with Covid-19. New England Journal of Medicine 384 795 807 10.1056/NEJMoa2031994 PMC7745180 33306283

[ref79] Marconi VC Ramanan AV de Bono S Kartman CE Krishnan V 2021 Efficacy and safety of baricitinib for the treatment of hospitalised adults with COVID-19 (COV-BARRIER): a randomised, double-blind, parallel-group, placebo-controlled phase 3 trial 10 00331 3 10.1016/S2213-2600(21)00331-3 PMC8409066 34480861

[ref80] Guimaraes PO Quirk D Furtado RH Maia LN Saraiva JF 2021 Tofacitinib in Patients Hospitalized with Covid-19 Pneumonia. New England Journal of Medicine 385 406 415 10.1056/NEJMoa2101643 PMC8220898 34133856

[ref81] Chen CX Wang JJ Li H Yuan LT Gale RP 2021 JAK-inhibitors for coronavirus disease-2019 (COVID-19): a meta-analysis Leukemia 35 2616 2620 33990684 10.1038/s41375-021-01266-6 PMC8119232

[ref82] Sparks JA Wallace ZS Seet AM Gianfrancesco MA Izadi Z 2021 Associations of baseline use of biologic or targeted synthetic DMARDs with COVID-19 severity in rheumatoid arthritis: Results from the COVID-19 Global Rheumatology Alliance physician registry Annals of the Rheumatic Diseases 80 1137 1146 34049860 10.1136/annrheumdis-2021-220418 PMC8172266

[ref83] Solimani F Meier K Ghoreschi K. 2021 Janus kinase signaling as risk factor and therapeutic target for severe SARS-CoV-2 infection European Journal of Immunology 51 1071 1075 33675065 10.1002/eji.202149173 PMC8250126

[ref84] Rodriguez-Garcia JL Sanchez-Nievas G Arevalo-Serrano J Garcia-Gomez C Jimenez-Vizuete JM 2021 Baricitinib improves respiratory function in patients treated with corticosteroids for SARS-CoV-2 pneumonia: an observational cohort study Rheumatology (Oxford) 60 399 407 33020836 10.1093/rheumatology/keaa587 PMC7665718

[ref85] Conti P Gallenga CE Tete G Caraffa A Ronconi G Agents 2020 How to reduce the likelihood of coronavirus-19 (CoV-19 or SARS-CoV-2) infection and lung inflammation mediated by IL-1 34 333 338 10.23812/Editorial-Conti-2 32228825

[ref86] Chen IY Moriyama M Chang MF Ichinohe T. Severe 2019 Acute Respiratory Syndrome Coronavirus Viroporin 3a Activates the NLRP3 Inflammasome 10 50 50 10.3389/fmicb.2019.00050 PMC6361828 30761102

[ref87] Zhao N Di B Xu LL 2021 The NLRP3 inflammasome and COVID-19: Activation, pathogenesis and therapeutic strategies Cytokine Growth Factor Reviews 61 2 15 34183243 10.1016/j.cytogfr.2021.06.002 PMC8233448

[ref88] Satis H Ozger HS Aysert Yildiz P Hizel K Gulbahar O 2021 Prognostic value of interleukin-18 and its association with other inflammatory markers and disease severity in COVID-19 137 155302 155302 10.1016/j.cyto.2020.155302 PMC7522034 33002740

[ref89] Opal SM Fisher CJ Dhainaut JF Vincent JL Brase R 1997 Confirmatory interleukin-1 receptor antagonist trial in severe sepsis: a phase III, randomized, double-blind, placebo-controlled, multicenter trial The Interleukin-1 Receptor Antagonist Sepsis Investigator Group. Critical Care Medicine 25 1115 1124 9233735 10.1097/00003246-199707000-00010

[ref90] Cavalli G De Luca G Campochiaro C Della-Torre E Ripa M 2020 Interleukin-1 blockade with high-dose anakinra in patients with COVID-19, acute respiratory distress syndrome, and hyperinflammation: a retrospective cohort study Lancet Rheumatology 2 e325 e331 32501454 10.1016/S2665-9913(20)30127-2 PMC7252085

[ref91] The CORIMUNO 2021 Effect of anakinra versus usual care in adults in hospital with COVID-19 and mild-to-moderate pneumonia (CORIMUNO-ANA-1): a randomised controlled trial Lancet Respiratory Medicine 9 295 304 33493450 10.1016/S2213-2600(20)30556-7 PMC7825875

[ref92] Kyriazopoulou E Huet T Cavalli G Gori A Kyprianou M 2021 Effect of anakinra on mortality in patients with COVID-19: a systematic review and patient-level meta-analysis Lancet Rheumatology 3 e690 e697 34396156 10.1016/S2665-9913(21)00216-2 PMC8352496

[ref93] Kyriazopoulou E Panagopoulos P Metallidis S Dalekos GN Poulakou G 2021 An open label trial of anakinra to prevent respiratory failure in COVID-19 10 10.7554/eLife.66125 PMC8034977 33682678

[ref94] Kyriazopoulou E Poulakou G Milionis H Metallidis S Adamis G 2021 Early treatment of COVID-19 with anakinra guided by soluble urokinase plasminogen receptor plasma levels: a double-blind, randomized controlled phase 3 trial 10 10.1038/s41591-021-01499-z PMC8516650 34480127

[ref95] Caricchio R Abbate A Gordeev I Meng J Hsue PY 2021 Effect of Canakinumab vs Placebo on Survival Without Invasive Mechanical Ventilation in Patients Hospitalized With Severe COVID-19: A Randomized Clinical Trial Journal of the American Medical Association 326 230 239 34283183 10.1001/jama.2021.9508 PMC8293025

[ref96] Deftereos SG Siasos G Giannopoulos G Vrachatis DA Angelidis C 2020 The GReek study in the Effects of Colchicine in COvid-19 complications prevention (GRECCO-19 study): rationale and study design Hellenic Journal of Cardiology 61 42 45 32251729 10.1016/j.hjc.2020.03.002 PMC7194546

[ref97] Tardif JC Kouz S Waters DD Bertrand OF Diaz R 2019 Efficacy and Safety of Low-Dose Colchicine after Myocardial Infarction New England Journal of Medicine 381 2497 2505 10.1056/NEJMoa1912388 31733140

[ref98] Lopes MI Bonjorno LP Giannini MC Amaral NB Menezes PI 2021 Beneficial effects of colchicine for moderate to severe COVID-19: a randomised, double-blinded, placebo-controlled clinical trial RMD Open 7 10.1136/rmdopen-2020-001455 PMC7868202 33542047

[ref99] Tardif JC Bouabdallaoui N Allier PL Gaudet D Shah B 2021 Colchicine for community-treated patients with COVID-19 (COLCORONA): a phase 3, randomised, double-blinded, adaptive, placebo-controlled, multicentre trial Lancet Respiratory Medicine 9 924 932 34051877 10.1016/S2213-2600(21)00222-8 PMC8159193

[ref100] 2021 Colchicine in patients admitted to hospital with COVID-19 (RECOVERY): a randomised, controlled, open-label, platform trial Lancet Respiratory Medicine 9 1419 1426 34672950 10.1016/S2213-2600(21)00435-5 PMC8523117

[ref101] Lien C H Lee MD Weng S L Lin CH Liu LY Meta-Analysis. Life 2021 Repurposing Colchicine in Treating Patients with COVID-19: A Systematic Review 11 864 864 10.3390/life11080864 PMC8398430 34440608

[ref102] Madrid-Garcia A Perez I Colomer JI Leon-Mateos L Jover JA 2021 Influence of colchicine prescription in COVID-19-related hospital admissions: a survival analysis Therapeutic Advances in Musculoskeletal Disease 13 10.1177/1759720X211002684 PMC8010810 33854571

[ref103] Kharouf F Ishay Y Kenig A Bitan M Ben-Chetrit E 2021 Incidence and course of COVID-19 hospitalizations among patients with familial Mediterranean fever Rheumatology (Oxford) 10 10.1093/rheumatology/keab577 PMC8344485 34293118

[ref104] Satis H Armagan B Bodakci E Atas N Sari A 2020 Colchicine intolerance in FMF patients and primary obstacles for optimal dosing Turkish Journal of Medical Sciences 50 1337 1343 32512676 10.3906/sag-2001-261 PMC7491296

[ref105] Haga S Yamamoto N Nakai-Murakami C Osawa Y Tokunaga K 2008 Modulation of TNF-alpha-converting enzyme by the spike protein of SARS-CoV and ACE2 induces TNF-alpha production and facilitates viral entry Proceedings of the National Academy of Sciences 105 7809 7814 10.1073/pnas.0711241105 PMC2409424 18490652

[ref106] Robinson PC Liew DFL Liew JW Monaco C Richards D Med 2020 The Potential for Repurposing Anti-TNF as a Therapy for the Treatment of COVID-19 1 90 102 10.1016/j.medj.2020.11.005 PMC7713589 33294881

[ref107] Feldmann M Maini RN Woody JN Holgate ST Winter G 2020 Trials of anti-tumour necrosis factor therapy for COVID-19 are urgently needed The Lancet 10 30858 8 10.1016/S0140-6736(20)30858-8 PMC7158940 32278362

[ref108] Kridin K Schonmann Y Solomon A Damiani G Tzur Bitan 2021 Mortality in Patients with Psoriasis Treated by Interleukin-17 Inhibitors Risk of COVID-19 Infection 10 1 28 10.1080/09546634.2021.1905766 33759683

[ref109] Avdeev SN Trushenko NV Tsareva NA Yaroshetskiy AI Merzhoeva ZM 2021 Anti-IL-17 monoclonal antibodies in hospitalized patients with severe COVID-19: A pilot study Cytokine 146 155627 155627 34237556 10.1016/j.cyto.2021.155627 PMC8253694

[ref110] Crotti C Biggioggero M Becciolini A Agape E Favalli EG 2019 Mavrilimumab: a unique insight and update on the current status in the treatment of rheumatoid arthritis Expert Opinion on Investigational Drugs 28 573 581 31208237 10.1080/13543784.2019.1631795

[ref111] Cremer PC Abbate A Hudock K McWilliams C Mehta J 2021 Mavrilimumab in patients with severe COVID-19 pneumonia and systemic hyperinflammation (MASH-COVID): an investigator initiated, multicentre, double-blind, randomised, placebo-controlled trial Lancet Rheumatology 3 e410 e418 33754144 10.1016/S2665-9913(21)00070-9 PMC7969143

[ref112] Temesgen Z Burger CD Baker J Polk C Libertin C 2021 Lenzilumab Efficacy and Safety in Newly Hospitalized Covid-19 Subjects: Results from the Live-Air Phase 3 Randomized Double-Blind Placebo-Controlled Trial 10

